# Association Between Menstrual Pain and Procedural Pain During Office Hysteroscopy: A 10‐Year Cohort Study

**DOI:** 10.1155/prm/8879788

**Published:** 2026-02-23

**Authors:** Tricia Dewi Anggraeni, Gerald Sebastian Davis, Fitriyadi Kusuma, Gatot Purwoto

**Affiliations:** ^1^ Department of Obstetrics and Gynecology, Gynecologic Oncology Division, Dr. Cipto Mangunkusumo National General Hospital, Faculty of Medicine University of Indonesia, Jakarta, Indonesia

**Keywords:** gynecology, hysteroscopy, menstruation, outpatients, pain

## Abstract

**Background:**

Office hysteroscopy (OH) is the standard diagnostic method for evaluating intrauterine and endocervical pathology, yet the fear of procedural pain remains a major barrier to patient acceptance. This study examined whether menstrual pain can be used as a clinical reference to anticipate OH procedural pain and explored other predictors of procedural discomfort.

**Methods:**

A historical cohort study was conducted among women undergoing OH for abnormal uterine bleeding or postmenopausal bleeding at a tertiary referral hospital between 2014 and 2024. All procedures were performed by a single experienced hysteroscopist using a 4.9‐mm vaginoscopic technique with preprocedural acetaminophen. Menstrual pain and OH procedural pain were assessed using a 0–10 visual analogue scale (VAS). Demographic and clinical variables were extracted from medical records. Associations with OH pain were evaluated using Mann–Whitney *U* tests and multivariable linear regression.

**Results:**

A total of 488 women were included (median age: 45 years and median BMI: 24.5 kg/m^2^). Median VAS for menstrual pain was 1.0 (interquartile range [IQR]: 0–3) and for OH pain was 0.0 (IQR: 0–2). Mean OH pain was lower than mean menstrual pain. In bivariate analyses, younger age, nulliparity, and higher menstrual pain were associated with higher OH pain. In multivariable analysis, menstrual pain and nulliparity remained independent predictors of higher OH procedural pain, while increasing age was associated with slightly lower pain.

**Conclusion:**

OH was generally well tolerated, with pain typically lower than menstrual pain. Although menstrual pain, parity, and age explained only a modest proportion of pain variability, menstrual pain history offers a simple and patient‐centered way to help clinicians set realistic expectations about procedural discomfort.

## 1. Introduction

Office hysteroscopy (OH) is widely used in contemporary gynecology because it enables direct visualization of the uterine cavity in a minimally invasive setting [1]. However, procedural pain remains a major determinant of patient acceptance and tolerance [2]. Even when the technique is optimized, pain perception varies substantially across individuals, and fear of pain may discourage patients from undergoing OH [2, 3].

Pain during OH is predominantly visceral and is typically attributed to stimulation during passage through the cervical canal, distension of the uterine cavity by the distension medium, and contact with the uterine walls [4]. Beyond procedural stimuli, the expected intensity of pain is strongly shaped by individual pain sensitivity and anticipatory factors, which makes preprocedural counselling challenging in routine practice [4–6].

Menstrual pain may offer a clinically meaningful reference point for expectation setting because it reflects individual variability in uterine visceral pain processing [7, 8]. Dysmenorrhea is most commonly linked to increased production of prostaglandins and related inflammatory mediators through the cyclooxygenase pathway, which promotes myometrial hypercontractility and uterine ischemia [9, 10]. Importantly, recurrent menstrual pain has been associated with sensitization processes (including viscero‐visceral and viscero‐somatic sensitization), whereby repeated visceral nociceptive input can lower pain thresholds and amplify responses to later uterine or pelvic visceral stimuli [11]. Given that OH pain is also generated by uterine and cervical visceral afferent activation during distension and mechanical stimulation, a history of more severe dysmenorrhea may plausibly indicate heightened susceptibility to OH‐related discomfort [12, 13].

Accordingly, this study aimed to evaluate the association between self‐reported menstrual pain severity and OH procedural pain and to assess additional clinical predictors, with the goal of supporting more individualized, evidence‐informed pain expectation counselling and management.

## 2. Material and Methods

This historical cohort study included 488 women who underwent OH at the Gynecologic Polyclinic of Cipto Mangunkusumo National General Hospital, Jakarta, Indonesia, between 2014 and 2024. Ethical approval was granted by the hospital’s Research Ethics Committee. Women referred for intrauterine evaluation due to abnormal uterine bleeding (AUB) or postmenopausal bleeding (PMB) were eligible for inclusion. Patients with contraindications to OH according to institutional protocols were excluded.

All procedures were performed according to a standardized institutional protocol by a single experienced gynecologic hysteroscopist with more than 500 prior procedures. Before hysteroscopy, all patients received oral acetaminophen 1000 mg. A vaginoscopic approach was used with a 4.9‐mm continuous‐flow Bettocchi office hysteroscope (Karl Storz, Tuttlingen, Germany) equipped with a 30°, 2.9‐mm rod lens optic. Normal saline was used as the distension medium and delivered via a pressure infusion bag to maintain an intrauterine pressure of approximately 70 mmHg. Intrauterine and endocervical findings were managed as clinically indicated. Procedures were classified as diagnostic (visual inspection and/or endometrial biopsy) or therapeutic (including endometrial ablation, endometrial wall resection, polypectomy, and intrauterine system insertion or retrieval).

Menstrual pain history was obtained by patient self‐report at the clinic visit prior to the hysteroscopy, using a standardized 0–10 visual analogue scale (VAS), where 0 indicated no pain and 10 indicated the worst pain imaginable. This value was recorded in the patient’s medical record as part of the preprocedural assessment. Procedural pain during OH was assessed using the same VAS immediately after completion of the hysteroscopy, before the patient left the examination room. Patients were instructed to rate pain attributable only to the hysteroscopic procedure itself.

VAS scores were categorized as no pain (0), mild pain (1–3), moderate pain (4–7), and severe pain (8–10). The hysteroscopist was not informed of the patients’ menstrual pain scores at the time of the procedure to minimize expectation‐related bias.

Demographic and clinical variables, including age, body mass index (BMI), parity, menopausal status, and procedure type (diagnostic or therapeutic), were extracted from medical records. All patient identifiers were removed and replaced with numerical codes to ensure confidentiality.

Statistical analyses were performed using IBM SPSS Statistics version 28.0. Menstrual pain and OH procedural pain were summarized as medians with interquartile ranges (IQRs) and means ± standard deviation (SD). Bivariate associations between OH pain and candidate predictors were evaluated using the Mann–Whitney *U* test. Variables of interest were then entered into a multivariable linear regression model to estimate independent associations with OH pain, expressed as beta coefficients and *p* values. A predicted‐value plot of OH pain by menstrual pain was generated and stratified by age group.

## 3. Results and Discussion

### 3.1. Results

This study indicates that the mean and median of menstrual pain is higher than OH procedural pain (2.04 ± 2.58 vs. 1.30 ± 2.12 and 1.0 [0.0, 3.0 vs. 0.0 [0.0, 2.0]). The largest group of pain intensity in both menstrual pain and OH procedural pain is no pain, followed by mild pain. Regarding the menstrual pain status, 209 patients (42.8%) reported no menstrual pain while 279 (57.2%) experienced pain. The OH procedural pain status shows that 275 (56.4%) patients experienced no pain, while 213 (43.6%) had pain (Table 1).

**TABLE 1:** Pain intensity of menstruation and OH procedure.

Pain (VAS)	Median (IQR)	Mean ± SD	None *n* (%)	Mild *n* (%)	Moderate–severe *n* (%)
Menstruation	1.0 (0–3)	2.04 ± 2.58	209 (42.8)	162 (33.2)	117 (24.0)
OH procedure	0.0 (0–2)	1.30 ± 2.12	275 (56.4)	156 (32.0)	57 (11.7)

The median age of participants was 45 years with IQR range of 36–53 years. The median BMI was 24.5 kg/m^2^, which is categorized as overweight according to the WHO Asia‐Pacific BMI Criteria. Most participants were parous (68.4%); 289 (59.2%) underwent therapeutic procedure rather than purely diagnostic procedures (Table 2).

**TABLE 2:** Potential predictors other than menstrual pain.

Predictor		Value
Age (years)^a^		45.0 (36–53)
BMI (kg/m^2^)^a^		24.5 (21.2–27.4)
Parity^b^	Nulliparous	154 (31.6)
	Parous	334 (68.4)
Menopausal status^b^	Menopause	173 (35.5)
	Nonmenopause	315 (64.5)
Procedure type^b^	Diagnostic	199 (40.8)
	Therapeutic	289 (59.2)

^a^Data reported as the median ± standard deviation.

^b^Data reported as *N* (%).

In the bivariate analysis, women younger than 45 years had significantly higher OH procedural pain scores compared with those aged 45 years or older (median 0.0 vs. 0.0, *p* = 0.016). Nulliparous women had higher OH procedural pain score than parous women (median: 1.0 vs. 0.0, *p* = 0.0008), and women with higher menstrual pain score (VAS ≥ 5) had significantly higher OH procedural pain score than those with menstrual VAS < 5 (median: 1.0 vs. 0.0, *p* = 0.0001). Procedure type (diagnostic vs. therapeutic), menopausal status (menopause vs., nonmenopause), and BMI category (< 25 vs. ≥ 25 kg/m^2^) were not significantly associated with OH procedural VAS (Table 3).

**TABLE 3:** Bivariate association between predictors with office hysteroscopy pain.

Predictor	Median OH procedural VAS (G1)	Median OH procedural VAS (G2)	*p* value^∗^
Age	0.0	0.0	0.016
BMI	0.0	0.0	0.79
Parity	1.0	0.0	0.0008
Menopausal status	0.0	0.0	0.072
Procedure type	0.0	0.0	0.74
Menstrual pain VAS	1.0	0.0	< 0.0001

^∗^Mann–Whitney U test.

In the multivariable linear regression model, higher menstrual pain score, younger age, and nulliparity were independently associated with higher OH procedural pain scores. Each one‐unit increase in menstrual pain score was associated with a 0.19‐point increase in OH procedural pain score (95% CI: 0.12–0.26, *p* < 0.001), each additional year of age with a 0.019‐point decrease (95% CI: −0.034–−0.004, *p* = 0.014), and nulliparity with a 0.42‐point higher OH procedural pain score compared with parous women (95% CI: 0.12–0.71, *p* = 0.005). The procedure type and therapeutic indication were not significant predictors after adjustment, and the overall model explained 8.5% of the variance in the OH procedural pain score (*R* ^2^ = 0.085).

**FIGURE 1:**
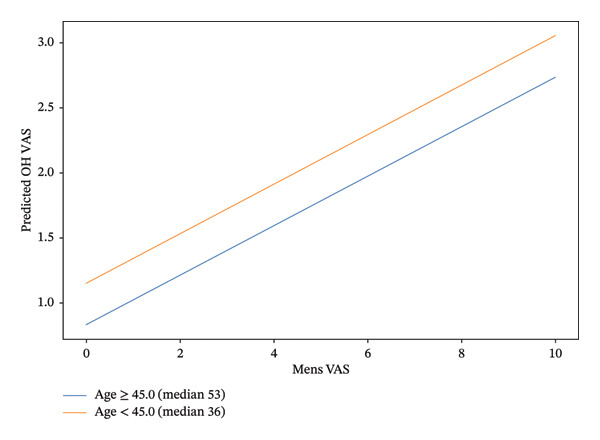
Predictive plot of the OH procedural pain score by the menstrual pain score stratified by age group.

A predictive model was made based on multivariable statistical analysis (Figure 1). The menstrual pain score showed a positive linear association with the predicted OH procedural pain score after adjustment for all covariates. When the model was stratified by age, the slope of the association between the menstrual pain score and predicted OH pain score was parallel in women aged < 45 years (median 36 years) and those aged ≥ 45 years (median 53 years), suggesting no evidence of statistical interaction between the age group and menstrual pain in determining predicted OH pain. Across the full range of menstrual pain score, the younger age group consistently had slightly higher predicted OH pain score than the older group. However, the highest score of the modeled OH pain score remained within the range interpreted as mild pain (VAS: 1–3).

### 3.2. Discussion

In this large outpatient cohort, procedural pain during OH was generally mild and lower than self‐reported menstrual pain. Median OH pain was 0.0 (IQR: 0.0–2.0) with mean VAS 1.30 ± 2.12, and the proportion of patients experiencing moderate‐to‐severe pain was relatively low. These findings are consistent with contemporary evidence that many women tolerate outpatient hysteroscopy well when standardized techniques and small‐diameter hysteroscopes are used even though pain remains an important determinant of patient experience and acceptance [14, 15].

Our results also demonstrate that higher menstrual pain (dysmenorrhea) was independently associated with higher procedural pain. This finding aligns with prior observations that women with a history of severe dysmenorrhea are more likely to report unacceptable pain following diagnostic hysteroscopy, suggesting that past visceral pain experiences may modulate subsequent pain responses [16, 17]. Although the specific neurophysiological mechanisms are not fully captured in clinical cohorts, this association may reflect shared visceral afferent pathways and individual differences in pain processing.

In addition to menstrual pain, parity and age were associated with OH procedural pain. Nulliparous women tended to report higher pain scores, consistent with other clinical analyses showing that parity influences tolerance to uterine distension and cervical passage [18]. Similarly, age has been variably associated with procedural pain, potentially reflecting differences in cervical compliance and pain perception across the reproductive lifespan. These findings corroborate recent work identifying patient‐related factors such as parity and anatomical characteristics such as cervical stenosis as predictors of discomfort during outpatient hysteroscopy [19, 20].

Despite these associations, the multivariable model explained only a modest proportion of pain variability (*R* ^2^ = 0.085), indicating that the majority of individual differences in pain experience remain unexplained by the variables included. This limited explained variance is expected given the multifactorial nature of pain perception, which encompasses psychological variables such as anxiety, pain catastrophizing, previous procedural experiences, and clinician–patient communication that were not available in this retrospective dataset.

Using menstrual pain as a practical reference for counseling may have clinical value, particularly in outpatient settings where detailed expectation management is challenging [21, 22]. Qualitative research has shown that patients commonly frame anticipated procedural pain relative to familiar experiences such as menstrual cramps, supporting the use of familiar pain references in preoperative discussion [23, 24]. Moreover, current clinical guidance on pain management for in‐office uterine procedures emphasizes individualized strategies rather than routine analgesic premedication, further highlighting the potential role of simple, patient‐centered predictors in expectation setting [25].

Although this study proposes a predictive model based on menstrual pain, age, and parity, it should be regarded as exploratory and hypothesis‐generating rather than a validated clinical tool. External validation in prospective cohorts, including diverse populations and additional patient‐reported factors such as baseline anxiety and pain coping styles, is necessary before the model can be adopted in routine practice.

This study has limitations inherent to its retrospective design and reliance on medical record data. Important psychological predictors known to influence pain perception were unavailable, and all procedures were performed by a single experienced operator, which may limit generalizability. Future research should involve multiple centers, broader patient populations, and standardized prospective assessment of both clinical and psychological predictors to refine and validate predictive models for procedural pain in OH (Table 4).

**TABLE 4:** Linear regression model of possible predictive factors associated with office hysteroscopy (OH) pain.

Predictor	β coefficient	95% CI	*p* value
Menstrual VAS	0.19	[0.12, 0.26]	< 0.001
Age	−0.019	[−0.034, −0.004]	0.014
Therapeutic	−0.11	[−0.48, 0.26]	0.56
Parity	0.42	[0.12, 0.71]	0.005
Intercept	1.79	[1.03, 2.56]	< 0.001
Model *R* ^2^	0.085	—	—

## 4. Conclusion

Most women experience less pain during OH than during their usual menstrual periods. Menstrual pain is the strongest clinical predictor of procedural discomfort and can be used as a simple, patient‐centered reference to set realistic expectations before hysteroscopy. Using this familiar benchmark may help reduce anxiety, improve patient preparedness, and support shared decision‐making for OH.

## Author Contributions

Tricia Dewi Anggraeni: conceptualization, methodology, resources, supervision, and writing–review and editing. Gerald Sebastian Davis: data curation, formal analysis, visualization, and writing–original drafts. Fitriyadi Kusuma and Gatot Purwoto: supervision, writing–review and editing.

## Funding

No funding was received for this research.

## Disclosure

All authors read and approved the final manuscript before submission.

## Ethics Statement

The study was approved by the Ethics Committee of the Faculty of Medicine, Universitas Indonesia–Cipto Mangunkusumo Hospital (no. KET‐242/UN2.F1/ETIK/PPM.00.02/2025) on March 10, 2025.

## Conflicts of Interest

The authors declare no conflicts of interest.

## Data Availability

The data used to support the findings of this study are available on request from the corresponding author.
